# *Chlamydia trachomatis* suppresses host cell store-operated Ca^2+^ entry and inhibits NFAT/calcineurin signaling

**DOI:** 10.1038/s41598-022-25786-y

**Published:** 2022-12-10

**Authors:** Nicholas B. Chamberlain, Zoe Dimond, Ted Hackstadt

**Affiliations:** Host-Parasite Interactions Section, Laboratory of Bacteriology, National Institute of Allergy and Infectious Diseases, National Institutes of Health, Hamilton, MT USA

**Keywords:** Cell biology, Microbiology

## Abstract

The obligate intracellular bacterium, *Chlamydia trachomatis*, replicates within a parasitophorous vacuole termed an inclusion. During development, host proteins critical for regulating intracellular calcium (Ca^2+^) homeostasis interact with the inclusion membrane. The inclusion membrane protein, MrcA, interacts with the inositol-trisphosphate receptor (IP_3_R), an ER cationic channel that conducts Ca^2+^. Stromal interaction molecule 1 (STIM1), an ER transmembrane protein important for regulating store-operated Ca^2+^ entry (SOCE), localizes to the inclusion membrane via an uncharacterized interaction. We therefore examined Ca^2+^ mobilization in *C. trachomatis* infected cells. Utilizing a variety of Ca^2+^ indicators to assess changes in cytosolic Ca^2+^ concentration, we demonstrate that *C. trachomatis* impairs host cell SOCE. Ca^2+^ regulates many cellular signaling pathways. We find that the SOCE-dependent NFAT/calcineurin signaling pathway is impaired in *C. trachomatis* infected HeLa cells and likely has major implications on host cell physiology as it relates to *C. trachomatis* pathogenesis.

## Main

The phylum *Chlamydiae* contains the human pathogens *Chlamydia trachomatis*, *C. psittaci*, and *C. pneumoniae*. Chlamydiae are obligate intracellular, gram negative bacteria that undergo a biphasic developmental cycle with the bacteria in either an infectious and metabolically constrained state, termed the elementary body (EB), or a metabolically active and replicative state that is non-invasive, and termed a reticulate body (RB)^1,2^. The intracellular development of chlamydiae occurs within a parasitophorous vacuole that is referred to as an inclusion^3^. *C. trachomatis* contains multiple serological variants that demonstrate specific organotropism and associated disease. Serovars A–C are associated with endemic, blinding trachoma, serovars D–K are responsible for the common sexually transmitted urogenital infections, and serovars L1–L3 are the causative agents of lymphogranuloma venereum, a more invasive disease^4–6^.

*C. trachomatis* utilizes a type III secretion system to deliver effectors across the inclusion membrane and into the cytosol of the host cell^7^. A subset of these T3SS effector proteins, termed Incs, localize to the inclusion membrane via a bi-lobed transmembrane domain^8,9^. Incs are oriented in the inclusion membrane in a manner that exposes them to the cytosol of the host cell^10,11^, enabling the Incs to interact with cytosol-exposed proteins^12^. While Incs are typically distributed relatively evenly around the inclusion membrane, a subset of Incs are localized to discrete, punctate sites on the membrane, termed microdomains, that are also enriched in cholesterol and active host Src-family kinases^13^. Currently, the *C. trachomatis* effectors CT101 (MrcA), CT147, CT222, CT223 (IPAM), CT224, CT228, CT232 (IncB), CT233 (IncC), CT288, and CT850 have been identified as Incs enriched in inclusion microdomains^9,13–15^. Early findings demonstrated microdomains function as hubs for interactions with the cytoskeleton and promote the positioning of the inclusion at the microtubule organizing center^13,16^. Microdomains also influence extrusion-based dissemination, a process in which all or part of an intact chlamydial inclusion is exocytosed from the host cell for dissemination to distal anatomic sites. The phosphorylation of myosin light chain 2 (MLC2) at the microdomain is a critical regulator of this dissemination process^14^, which occurs via ionized calcium (Ca^2+^)-dependent pathways^17^.

Cytosolic Ca^2+^ acts as a second messenger for a number of pathways in eukaryotes influencing fertilization, embryonic axis formation, cell differentiation, cell proliferation, transcription factor activation, and cell fate decisions^18,19^. Cytosolic Ca^2+^ is tightly regulated to ensure these pathways are activated only when required. This homeostatic regulation of cytosolic Ca^2+^ concentration is achieved through Ca^2+^ pumps, exchangers, channels, buffering proteins, and sensors to control Ca^2+^ movement across the plasma membrane and the membranes of intracellular Ca^2+^ stores^20^. In excitable and non-excitable cells, store-operated Ca^2+^ entry (SOCE) is a major regulator of Ca^2+^ homeostasis^21^. The SOCE process is initiated by the depletion of endoplasmic reticulum (ER) Ca^2+^. The reduction in ER luminal Ca^2+^ is sensed by stromal interaction molecule 1 (STIM1) and subsequently initiates a STIM1 conformational change enabling STIM1 to interact with Orai1 to generate Ca^2+^ release-activated Ca^2+^ (CRAC) influx channels at the plasma membrane. The STIM1-CRAC channel interaction opens the channel resulting in an ingress of extracellular Ca^2+^ into the cytosol^22^. This influx of extracellular Ca^2+^ into the cytosol refills Ca^2+^ stores via the sarco-endoplasmic reticulum Ca^2+^ ATPase (SERCA)^23^ and is a signal for select Ca^2+^-dependent pathways.

The *C. trachomatis* inclusion interacts with the host ER, a major intracellular Ca^2+^ store, through multiple interactions including the inclusion membrane protein IncD interaction with ceramide transfer protein (CERT)^24^, the direct interaction of IncV with VAP^25^, the interaction of STIM1 with the inclusion membrane by an unknown mechanism^17,26^, and the interaction of MrcA with the inositol triphosphate receptor (IP_3_R)^17^. STIM1 and IP_3_R localization to microdomains of the inclusion membrane^17,26^ demonstrate that regulators of Ca^2+^ homeostasis are recruited during chlamydial development. Therefore, we investigated if chlamydial development disrupts host cell Ca^2+^ homeostasis. Here we provide evidence that SOCE is impaired by the midpoint of the chlamydial developmental cycle and the SOCE-inducible NFAT/calcineurin signaling pathway is concurrently abrogated, which could have major implications on host cell physiology.

## Results

### Store-operated Ca^2+^ entry is impaired by mid-cycle of the ***C. trachomatis*** developmental cycle

**Figure 1:**
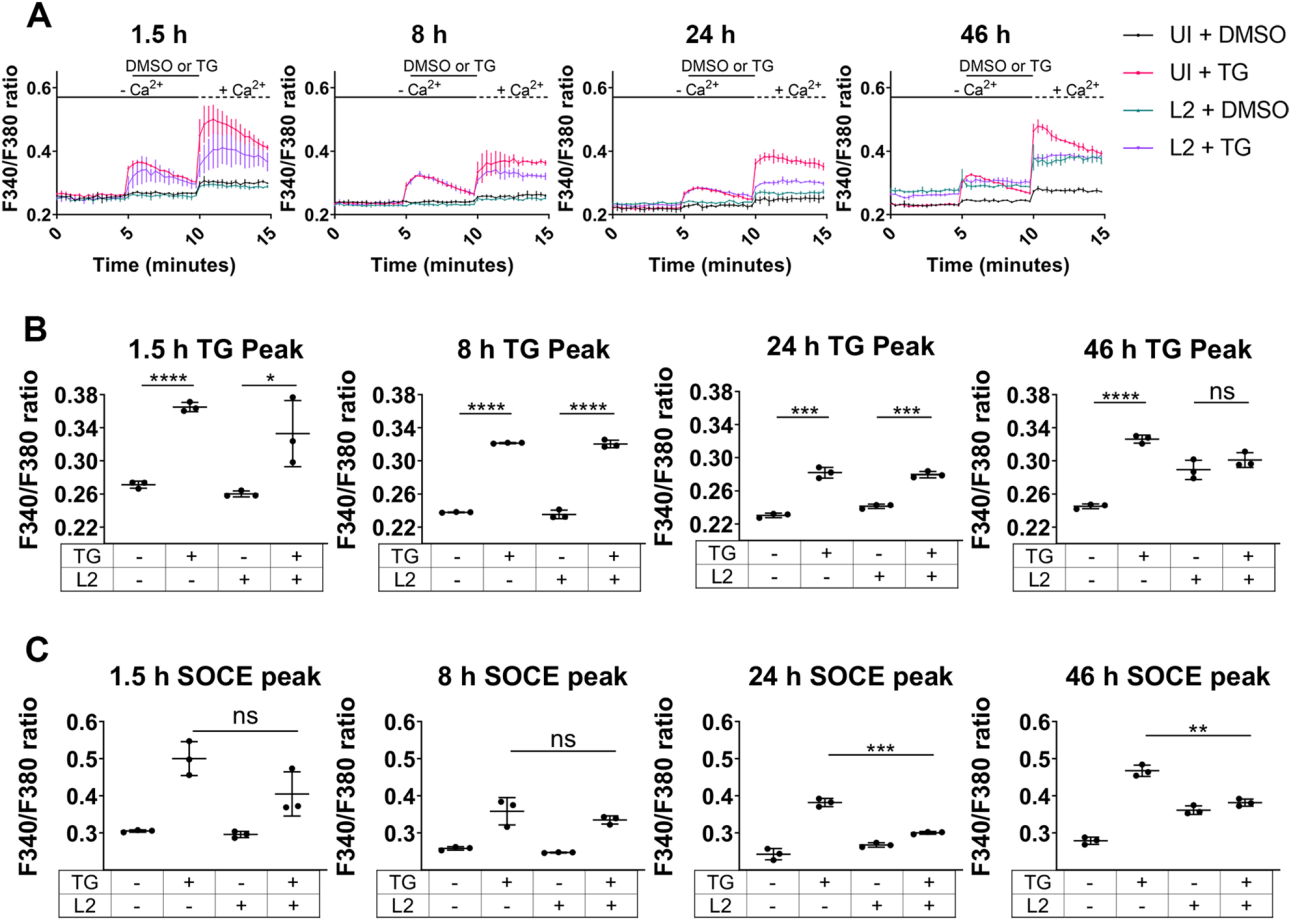
*C. trachomatis* impairs host cell store-operated calcium entry (SOCE) by a mid-cycle developmental time point. (**a**) Ca^2+^ re-addition assays were performed with Fura-2, AM to assess [Ca^2+^]_i_ changes in HeLa cells infected with *C. trachomatis* L2 or uninfected. Thapsigargin (TG) was used to induce ER Ca^2+^ depletion and subsequent SOCE. DMSO was used as a vehicle control. A ratiometric assessment of Fura-2, AM fluorescence at 340 nm and 380 nm was taken to calculate the relative change in [Ca^2+^]_i_. (**b**) The peak Ca^2+^ efflux from the ER induced by TG for each time point was measured. The TG treatment was compared to DMSO for either uninfected or infected cells. Student’s T-test was used to compare the DMSO to the TG treatment, n = 3. (**c**) Peak SOCE for each time point was calculated. Student’s T-test was used to compare the uninfected TG treated condition to the infected TG treated conditions, n = 3. The SOCE peak for TG-treated samples was not significantly different between infected and uninfected cells at 1.5 hpi (*p* = 0.0929) or 8 hpi (*p* = 0.3491), however, it was significantly reduced in infected cells at the mid-cycle time point (24 hpi) (*p* = 0.002) and the 46 hpi time point (*p* = 0.0012). Data are presented as mean ± SEM.

To investigate the impact of chlamydial infections upon intracellular Ca^2+^ mobilization, the ratiometric Ca^2+^ indicator, Fura-2, AM was used to assess changes in host cell intracellular Ca^2+^ concentration ([Ca^2+^]_i_). HeLa cells either uninfected or infected with *C. trachomatis* L2 were loaded with Fura-2, AM at the desired time post infection. The binding of Ca^2+^ to Fura-2, AM induces a shift in its fluorescence excitation from 380 to 340 nm. Therefore, a 340 nm/380 nm fluorescence ratio of Fura-2, AM was used to determine relative changes in Ca^2+^ concentration. A Ca^2+^ re-addition assay^27^ was used to obtain ratiometric measurements in a resting state, during induced ER Ca^2+^ leakage, and throughout SOCE. Following a resting state baseline reading in a Ca^2+^-free Ringer’s solution, cells were incubated with Ca^2+^-free Ringer’s solution containing either thapsigargin (TG) or the vehicle control, DMSO. TG is an inhibitor of the SERCA Ca^2+^ pump responsible for mobilizing Ca^2+^ from the cytosol into the lumen of the ER or sarcoplasmic reticulum. TG thus causes an increase in cytosolic Ca^2+^ by impairing ER Ca^2+^ uptake while ER Ca^2+^ depletion occurs via passive ER Ca^2+^ leakage through ER translocon complexes^28,29^. When TG treated and DMSO control HeLa cells were moved to a Ca^2+^-containing Ringer’s solution, a distinctive increase in [Ca^2+^]_i_ was detected in TG-treated, but not DMSO treated, cells indicating that Ca^2+^ depletion of the ER resulted in the activation of SOCE (Fig. 1a). A STIM1 siRNA knockdown was performed to verify this methodology (Extended Data Fig. 1).

To interrogate changes in Ca^2+^ mobilization during *C. trachomatis* development, the Fura-2, AM Ca^2+^ re-addition assay was performed with HeLa cells infected with *C. trachomatis* L2 at early- (1.5 hpi), early-to-mid- (8 hpi), mid- (24 hpi), and late- (46 hpi) cycle developmental time points (Fig. 1a). TG induced a significant increase in cytosolic Ca^2+^ at all time points for uninfected and infected cells, except for 46 hpi, indicating TG triggered ER Ca^2+^ egress in infected and uninfected cells (Fig. 1b). While the SOCE peak was not significantly different between infected and uninfected cells at 1.5 hpi or 8 hpi, it was significantly depressed in infected cells at the mid-cycle time point (24 hpi) and the 46 hpi time point, indicating *C. trachomatis* impairs SOCE of the host cell by a mid-cycle developmental timepoint (Fig. 1c). At 46 hpi, the baseline level of F340/F380 was elevated in the infected vs the uninfected. The likely interpretation of this would be that membranes were compromised due to lysis resulting in elevated [Ca^2+^]_i_ at this timepoint which is near the end of the *C. trachomatis* developmental cycle, and may explain why TG-induced ER Ca^2+^ egress and SOCE at this time point were impaired.

Intracellular pathogens depend upon viability of the host cell to complete their intracellular development^2^. We reconfirmed the viability of *C. trachomatis* infected cells at each of the time points analyzed. There was no significant difference in viability of uninfected versus infected cells at 8 h or 24 h post-infection. Viability was 89.3% ± 1.6% in uninfected versus 91.5% ± 2.4% (Mean ± SD; n = 6) in infected cells at 8 h post-infection and 90.8% ± 2.7% in uninfected versus 93.7% ± 2.0% in infected cells (Mean ± SD; n = 6) at 24 h post-infection. Viability dropped slightly in infected cells at 46 h post-infection from 88.0% ± 3.3% (Mean ± SD; n = 6) in uninfected to 80.7% ± 1.9% (Mean ± SD; n = 6) in infected cells (*p* < 0.013).

### Verification of *C. trachomatis*-suppressed SOCE using single-cell analysis

The Fura-2, AM-based method measured changes in cytoplasmic Ca^2+^ at the cell population level. To assess the influence of *C. trachomatis* serovar L2 on host cell Ca^2+^ mobilization at a single cell level, we used the Ca^2+^ indicator Fluo-4, AM with live-cell microscopy to determine changes in [Ca^2+^]_i_ in infected and uninfected cells. To quantify the normalized relative change in Fluo-4, AM fluorescence intensity (F) for each cell, ΔF/F_0_ was calculated. The baseline resting state fluorescence (F_0_) was the average of the first four mean intensity measurements in Ca^2+^-free Ringer’s solution, and ΔF = F − F_0_ was used to calculate the change in fluorescence.

**Figure 2:**
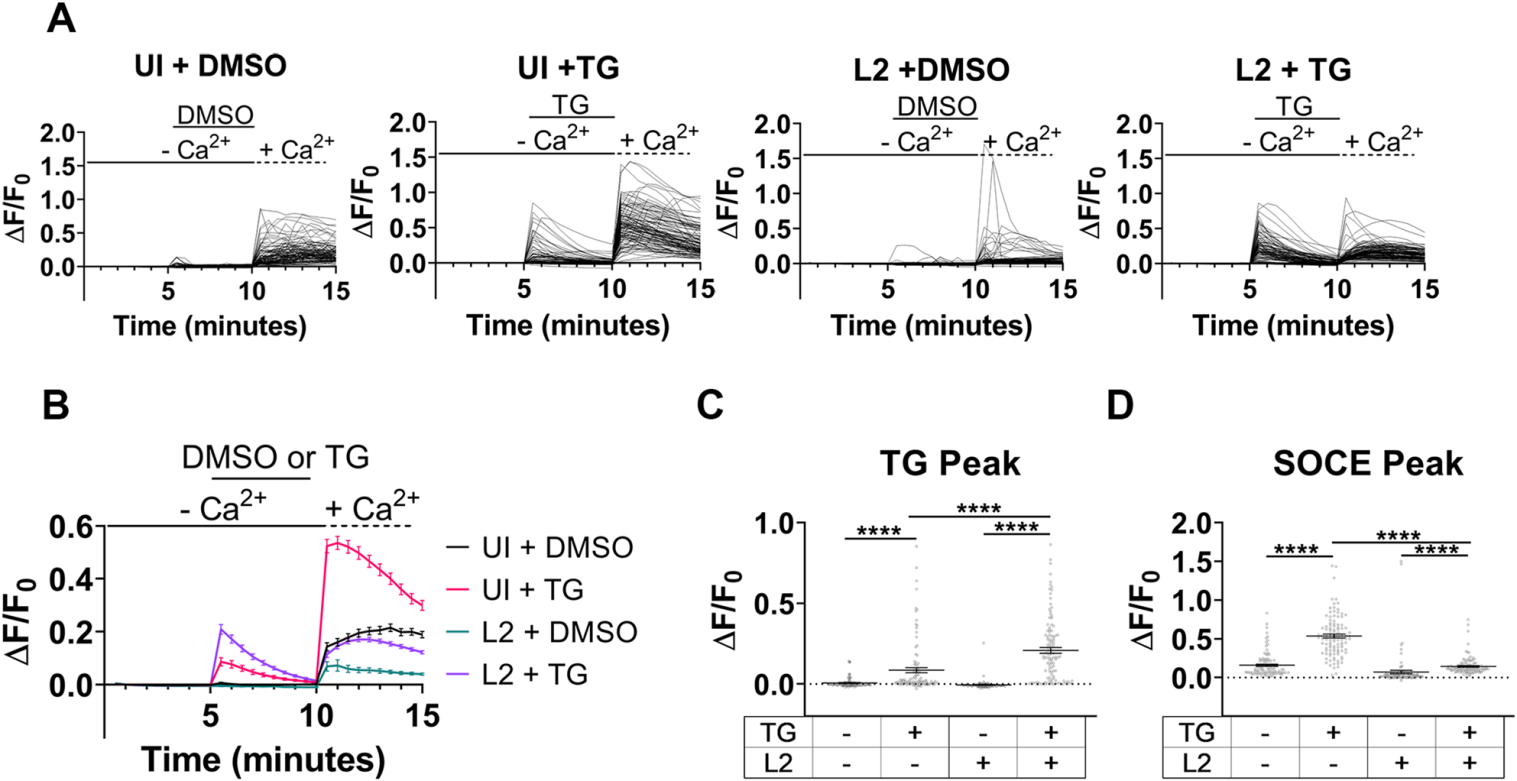
Fluo-4 assessment of *C. trachomatis* suppression of host cell SOCE. Fluo-4, AM, was used with live-cell microscopy to assess cytosolic Ca^2+^ mobilization in uninfected and *C. trachomatis* L2-infected cells. (**a**) Single-cell analysis of the relative change in Fluo-4 fluorescence during Ca^2+^ re-addition assay was performed for uninfected and *C. trachomatis*-infected cells at a mid-cycle developmental timepoint. For each condition, ≥ 39 cells were analyzed. (**b**) The mean relative change in Fluo-4 fluorescence was calculated for each condition (**a**). (**c**) The relative change in Fluo-4 fluorescence was assessed at the peak TG-induced ER Ca^2+^ egress. (**d**) The relative change in Fluo-4 fluorescence was calculated for peak SOCE. A Kruskal–Wallis test was performed with Dunn’s post-hoc multiple comparisons test to compare the conditions (**c**, **d**). Comparisons denoted with **** have a *p* value < 0.0001 and ns represents no significant difference. Data (**b–d**) are presented as mean ± SEM.

Analysis of [Ca^2+^]_i_ mobilization in uninfected and *C. trachomatis*-infected cells was performed at a mid-cycle (24 h) developmental timepoint. In uninfected and infected HeLa cells treated with the DMSO carrier, stochastic Ca^2+^ elevations were observed in a small subset of cells, and when DMSO-treated control and infected cells were placed in Ca^2+^-Ringer’s solution, there was a modest increase in [Ca^2+^]_i_ (Fig. 2a). When cells were treated with TG, a dramatic increase in ΔF/F_0_ was observed for both uninfected and infected cells, indicating the TG treatment mobilized Ca^2+^ from the ER into the cytoplasm. The transition of TG-treated cells to Ca^2+^-containing Ringer’s solution resulted in a striking increase in ΔF/F_0_ associated with SOCE in uninfected but not infected cells (Fig. 2a). The mean of the single-cell measurements provides a visualization of the single cell Ca^2+^ flux trends for each condition (Fig. 2b). TG-treated, *C. trachomatis*-infected cells demonstrated a significant increase in relative fluorescence of the Fluo-4, AM indicator compared to uninfected TG-treated cells (Fig. 2c). At 44 hpi, there was no significant difference in the TG peak between TG-treated uninfected and infected cells. (Extended Data Fig. 2a–c). These results indicate that TG induces Ca^2+^ egress from the ER of uninfected and infected cells.

The SOCE of uninfected and infected cells was also assessed using the Fluo-4, AM indicator. The mean ΔF/F_0_ at the SOCE peak for HeLa cells infected with *C. trachomatis* serovar L2 was severely reduced compared to uninfected cells. Furthermore, 53% of the uninfected, TG-treated cells had a ΔF/F_0_ greater than 0.5 compared with only 3% infected cells (Fig. 2d). Additionally, uninfected and infected DMSO treated cells had 5% and 2% of cells, respectively, with a ΔF/F_0_ of greater than 0.5 (Fig. 2d). At 44 hpi, infected cells had a significantly reduced ΔF/F_0_ mean at the SOCE peak compared to uninfected, and only 3% of the cells had a SOCE peak greater than 0.5 in the infected and TG-induced condition compared to 37% in the uninfected and TG-induced condition (Extended Data Fig. 2d). Collectively, the Fluo-4, AM single-cell analysis demonstrated that SOCE of the host cell is impaired by mid-cycle and remains suppressed at later developmental time points.

### Genetically encoded Ca^2+^ indicator confirms SOCE inhibition in ***C. trachomatis*** infected cells

The genetically encoded Ca^2+^ indicator (GECI), GCaMP6m, was used to corroborate the results of the Fura-2, AM and Fluo-4, AM based quantification of [Ca^2+^]_i_ mobilization in uninfected and *C. trachomatis*-infected cells. The GCaMP family of GECIs are single fluorescent protein indicators that operate by utilizing a circularly permuted GFP linked to calmodulin and the Ca^2+^-dependent calmodulin-interacting peptide M13 from myosin light chain kinase (MLCK). Ca^2+^ binding induces a conformational change in the circularly permuted GFP causing a large increase in fluorescence intensity^30^. Utilizing GCaMP6m, a relative change in GFP fluorescence can be used to determine changes in [Ca^2+^]_i_. *C. trachomatis* L2 expressing mScarlet permitted visualization of chlamydial inclusions during imaging. HeLa cells infected with mScarlet *C. trachomatis* L2 and transfected with pN1-GCaMP6m-XC were tested to assess Ca^2+^ mobilization.

**Figure 3:**
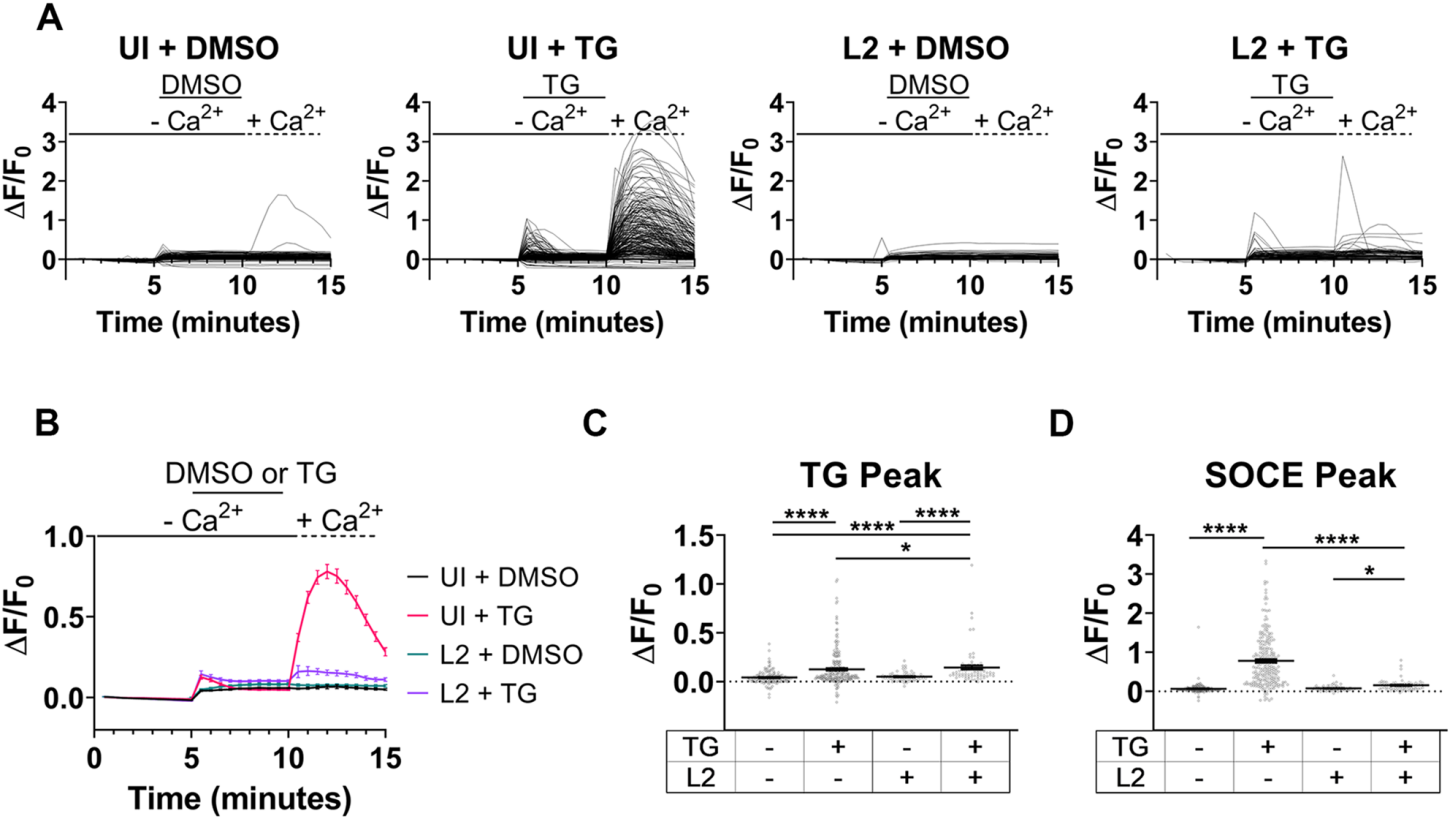
Genetically encoded Ca^2+^ indicator verification of impaired SOCE of host cell. Live-cell microscopy with the genetically encoded Ca^2+^ indicator, GCaMP6m, was used to measure changes in [Ca^2+^]_i_. (**a**) Relative change in GCaMP6m fluorescence was calculated throughout Ca^2+^ re-addition assay for individual HeLa cells at a mid-cycle (24 h) developmental timepoint. For each condition, ≥ 67 cells were analyzed. (**b**) The mean relative change of GCaMP6m fluorescence was measured from the single-cell analysis (**a**). (**c**) The relative change in GCaMP6m fluorescence at peak TG-induced ER Ca^2+^ efflux was calculated. (**d**) The relative change in GCaMP6m fluorescence was measured at peak ΔF/F_0_ during SOCE. Because GCaMP6m measurements were a non-Gaussian data set, a Kruskal–Wallis test was performed with Dunn’s post-hoc multiple comparisons test to compare the conditions (**c**, **d**). Comparisons denoted with **** have a *p* value < 0.0001 and * have a *p* value < 0.05. Data (**b–d**) are presented as mean ± SEM.

The single-cell and mean analysis of the relative fluorescence change of GCaMP6m demonstrated similar trends as the Fluo-4 analysis with SOCE of infected cells impaired relative to uninfected cells (Fig. 3a, b). The single-cell analysis at the TG peak indicated that both uninfected and infected cells had elevated fluorescence upon TG addition compared to the DMSO vehicle control (Fig. 3c). The single-cell analysis of the SOCE peak indicated that uninfected cells treated with TG had an increased relative change in GCaMP6m fluorescence compared to the DMSO control, however, there was no significant difference in the SOCE peak between the 24 hpi cells treated with DMSO and TG (Fig. 3d). The GCaMP6m analysis of HeLa cells at a mid-to-late developmental cycle timepoint, 36 hpi, with *C. trachomatis* serovar L2 demonstrated that SOCE was suppressed in infected cells (Extended Data Fig. 3). The Fura-2, Fluo-4, and GCaMP6m Ca^2+^ indicators collectively demonstrated that SOCE is impaired in HeLa cells infected with *C. trachomatis* L2 by mid-cycle developmental time point.

To determine if a urogenital *C. trachomatis* serovar also impairs SOCE of the host cell, the Ca^2+^ re-addition assay was performed using GCaMP6m in HeLa cells infected with *C. trachomatis* serovar D at a chlamydia developmental midpoint (24 h). Similar to the *C. trachomatis* L2 results, *C. trachomatis* D impaired SOCE of the host cell by the mid-cycle developmental timepoint (Extended Data Fig. 4).

### *C. trachomatis* prevents SOCE-induced NFAT nuclear localization

**Figure 4:**
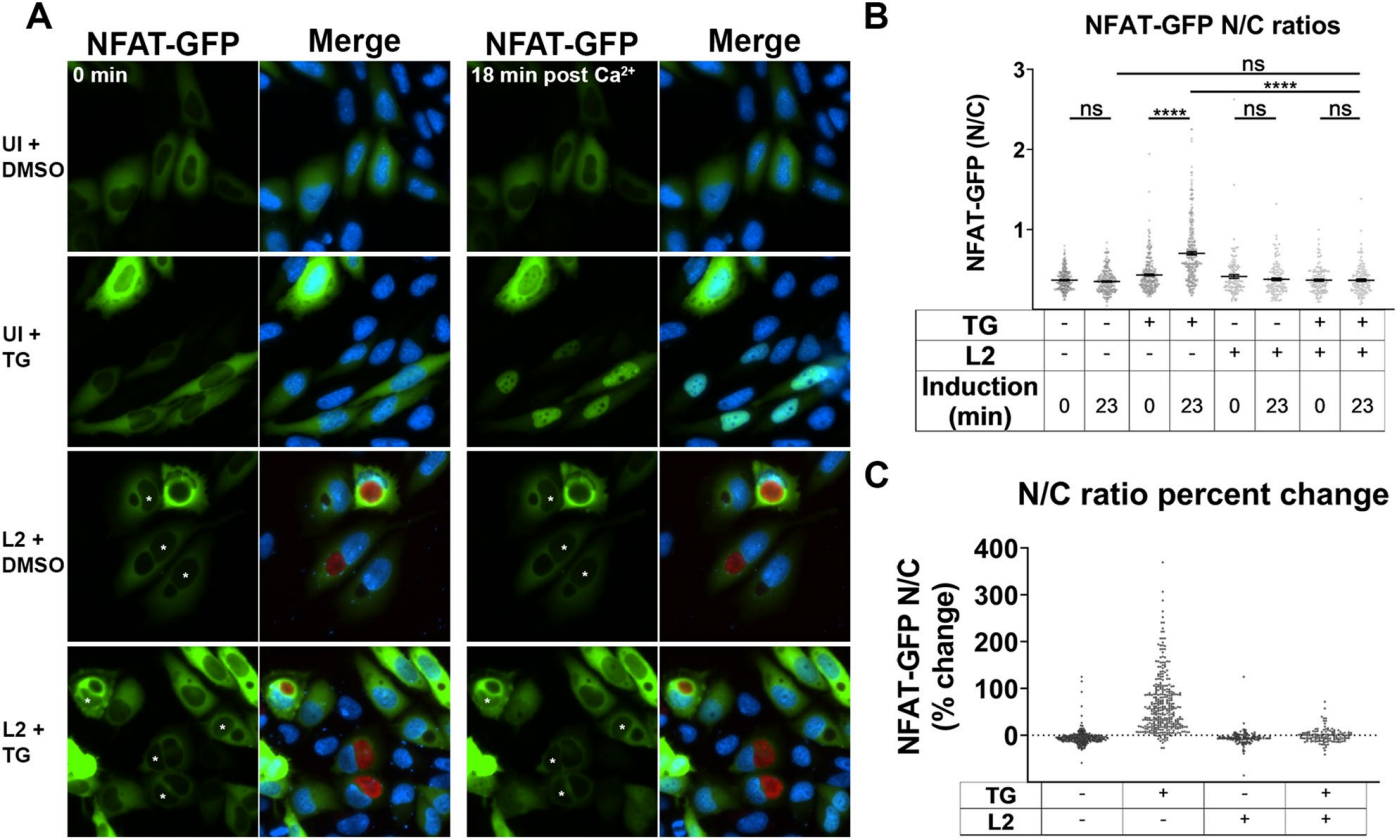
NFAT nuclear localization is diminished in *C. trachomatis*-infected cells. Nuclear translocation of NFAT-GFP was assessed in *C. trachomatis* L2 infected and uninfected HeLa cells. (**a**) Cells expressing NFAT-GFP were induced with thapsigargin (TG) to trigger SOCE or treated with the DMSO vehicle control for 5 min. Cells were imaged at 0 min post-addition of TG or DMSO, and then imaged at 18 min after administering Ca^2+^-containing Ringer’s solution. Asterisks denote the nuclei of *C. trachomatis*-infected cells. (**b**) Single-cell analysis of the NFAT-GFP nuclear-to- cytoplasmic mean fluorescence ratio (N/C) was calculated for each cell immediately following TG or DMSO treatment and following the 18 min Ca^2+^ incubation. For each condition, ≥ 134 cells were analyzed. A Kruskal–Wallis test was performed with Dunn’s post-hoc multiple comparisons test. Comparisons denoted with **** have a *p* value < 0.0001 and ns represents no significant difference. The data are presented as mean ± SEM. (**c**) The percent change in NFAT-GFP N/C ratio was calculated from the N/C ratios before and after induction (**b**) for each individual cell.

Extended increases in [Ca^2+^]_i_ caused by SOCE result in the activation of various Ca^2+^-dependent pathways. To gain insights into physiological consequences of impaired SOCE in *C. trachomatis* infected cells, a specific SOCE-dependent pathway was investigated. Sustained elevated [Ca^2+^]_i_ activates the calcineurin-NFAT signaling pathway via the Ca^2+^-mediated binding of calmodulin to calcineurin, the calcineurin-dependent dephosphorylation of the NFAT transcription factor to expose its nuclear localization signal, and the subsequent cytosol-to-nucleus translocation of NFAT. We investigated the SOCE-induced nuclear localization of NFAT1 in *C. trachomatis*-infected cells at the mid-cycle time point to determine if a SOCE-dependent pathway is abrogated during *C. trachomatis* infection. HeLa cells infected with mScarlet-expressing *C. trachomatis* L2 were transfected with the HA-NFAT1(4-460)-GFP plasmid. A pilot study indicated that the optimal time following TG treatment and Ca^2+^ incubation for NFAT-GFP nuclear translocation in HeLa cells was 18 min post-Ca^2+^ addition (Supplementary Video 1). Therefore, imaging for NFAT-GFP nuclear translocation was performed immediately following TG or DMSO treatment, and again at 18 min post-Ca^2+^ incubation. Imaging of NFAT-GFP expressing HeLa cells treated with DMSO demonstrated no noticeable change in nuclear NFAT-GFP fluorescence following the 18 min Ca^2+^ incubation, while the TG treatment caused a dramatic increase in nuclear NFAT-GFP following the Ca^2+^ incubation (Fig. 4a). However, neither the DMSO nor TG treatment caused a noticeable increase of nuclear NFAT-GFP in *C. trachomatis*-infected cells at 24 hpi (Fig. 4a). A representative time course video of NFAT-GFP-expressing HeLa cells infected with mScarlet *C. trachomatis* L2 did not demonstrate NFAT-GFP nuclear localization in the presence of TG, however, NFAT-GFP-expressing cells in the same field of view without a chlamydial inclusion showed nuclear translocation of NFAT-GFP (Supplementary Video 2).

Image analysis was performed to calculate the NFAT-GFP nuclear-to-cytoplasmic fluorescence ratio (N/C) for each condition. The NFAT-GFP N/C was measured as the mean fluorescence intensity of nuclear NFAT-GFP / the mean fluorescence intensity of cytoplasmic NFAT-GFP for individual cells. Cells with an observable inclusion containing mScarlet *C. trachomatis* were calculated for infected cells. The only significant difference identified between the pre- and post-incubation with Ca^2+^-containing Ringer’s solution was for the uninfected + TG condition, which demonstrated a substantial increase in the NFAT-GFP N/C ratio following the 18 min Ca^2+^ incubation (*p* value < 0.0001). No significant difference in NFAT-GFP N/C was observed for *C. trachomatis*-infected cells treated with TG post-Ca^2+^ incubation. (Fig. 4b). To visualize how the NFAT-GFP N/C ratio changed from post-TG or -DMSO treatment to post-Ca^2+^ incubation for each cell, a percent change in NFAT-GFP N/C ratio was calculated per cell (Fig. 4c). Collectively, the NFAT-GFP N/C ratio assessment demonstrated a severe reduction in NFAT-GFP nuclear localization in *C. trachomatis* L2 infected HeLa cells when induced to undergo SOCE.

## Discussion

We examined Ca^2+^ dynamics in *C. trachomatis*-infected cells and demonstrate that *C. trachomatis* inhibits SOCE of the host cell by a mid-cycle (24 h) developmental time point. This inhibition of SOCE was confirmed using three independent methods of intracellular Ca^2+^ quantitation. High concentrations of intracellular Ca^2+^ resulting from SOCE induction can act as a second messenger to activate numerous signaling pathways^18,31^. Among the pathways activated is the calcineurin/NFAT pathway. Sustained high concentrations of intracellular Ca^2+^ activates the phosphatase calcineurin which, in turn, dephosphorylates the cytoplasmic components of the NFAT transcription complex to trigger NFAT translocation into the nucleus^32^. NFAT transcription complexes regulate genes encoding immunomodulatory proteins or involved in developmental cellular differentiation^19,33,34^. *C. trachomatis* inhibition of SOCE had the downstream effect of inhibiting the calcineurin/NFAT pathway, thus providing a biological confirmation of the intracellular Ca^2+^ quantitation. The impairment of NFAT1 nuclear translocation demonstrate a specific signaling pathway that is affected by suppressing SOCE in *C. trachomatis* infected cells and likely has a multifaceted impact on host cell physiology and chlamydial pathogenesis.

At the end of their developmental cycle, chlamydiae are released by one of two distinct mechanisms for infection of adjacent cells and subsequent cycles of infection. Infectious EBs are released either by lysis of the infected cell, or intact or partially intact membrane bound inclusions are released by a process known as extrusion^35^. The cellular requirements for the two different release mechanisms are unique. Myosin II and Ca^2+^ are essential for chlamydial extrusion-based dissemination^14,17,35^. Ca^2+^ appears to be an important determinant for chlamydial release mechanism from the host cell, however, the suppression of SOCE indicates that other sources of Ca^2+^ must be utilized. The lack of synchrony and the fact that all infected cells do not undergo extrusion may present challenges in characterizing the formation of Ca^2+^ microenvironments near the inclusion microdomain.

**Figure 5:**
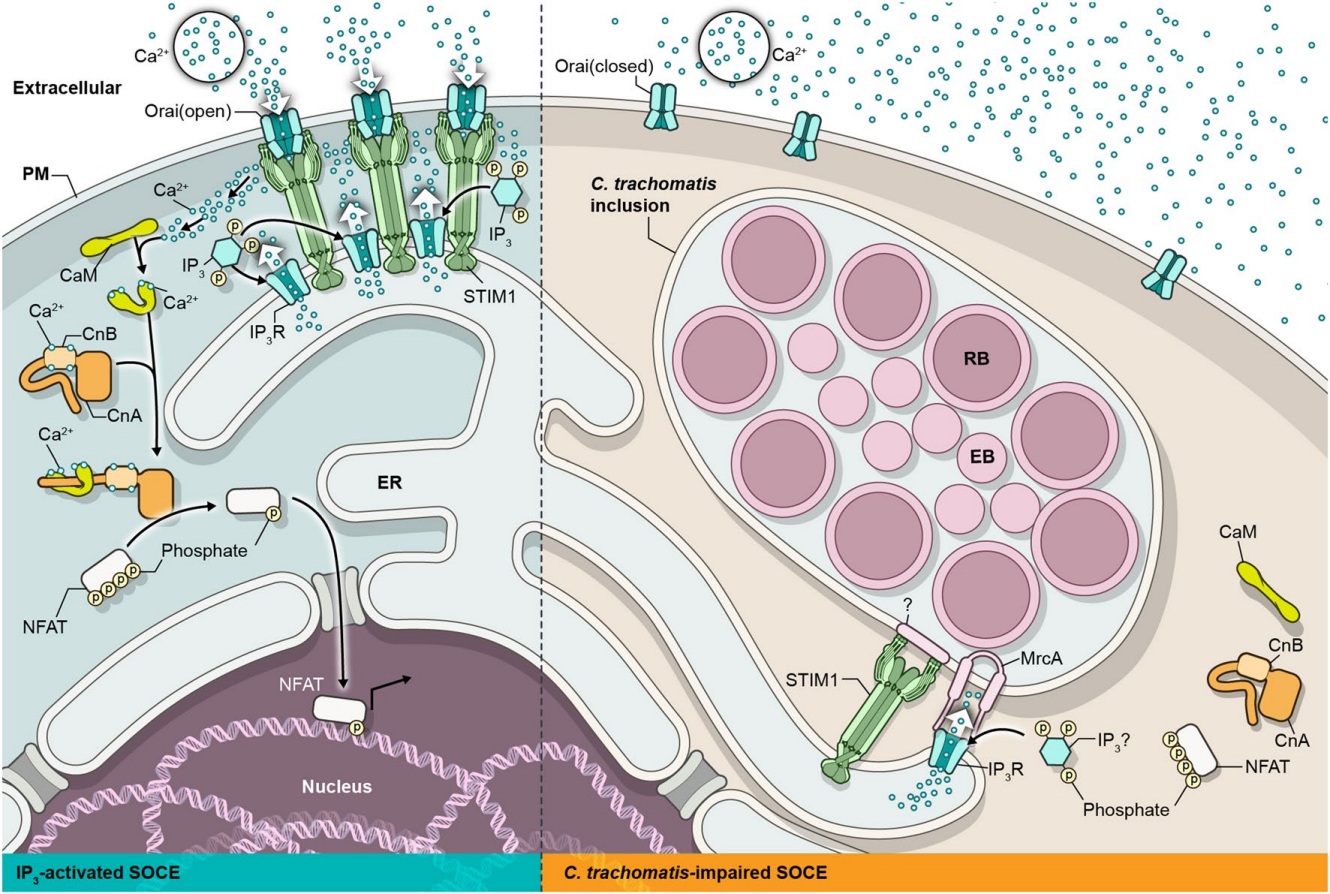
Working model for *C. trachomatis* impaired SOCE. Typical NFAT mobilization induced by SOCE in uninfected cells (left side) compared to a working model for *C. trachomatis* impaired SOCE (right side) and subsequent inhibition of calcineurin/NFAT signaling of the host cell. See text for details. The figure was created by the NAID Visual and Medical Arts Unit of the Research Technologies Branch as part of their official duties using Adobe Illustrator v26.0 https://www.adobe.com/products/illustrator.html.

Although the detailed mechanisms of chlamydial inhibition of SOCE remain unknown, at least two host components involved in calcium homeostasis are inappropriately localized in *C. trachomatis* infected cells. STIM1 and IP_3_R are recruited to and enriched in microdomains on the inclusion membrane^17,26^. IP_3_R is recruited to the inclusion membrane through interactions with the chlamydial protein, MrcA^17^. ATP-induced IP_3_R activation in *C. trachomatis* infected cells^36,37^ suggests MrcA does not have an inhibitory effect on IP_3_R. The mechanism of STIM1 recruitment is unknown though STIM1 is known to form complexes with IP_3_R^38^. Although STIM1 is recruited to the chlamydial inclusion, Orai1 was not and remained localized to the host plasma membrane^26^. A model for possible interactions and events associated with inhibition of SOCE and NFAT translocation is depicted in Fig. 5.

TG treatment induces puncta formation and co-localization of Orai1 and STIM1 in uninfected cells^39^. Puncta formation was previously observed in cells overexpressing fluorescent protein tagged STIM1 and Orai1^26^. While different pools of mCherry-STIM1 localized to the inclusion or the ER, Orai1-GFP did not associate with the inclusion membrane. When these cells were induced with TG to undergo SOCE, the inclusion membrane-associated mCherry-STIM1 remained at the inclusion, but the pool of ER-distributed mCherry-STIM1 demonstrated punctate staining indicative of ER-PM junctions necessary for the formation of CRAC channels. This suggested that an unknown chlamydial factor retains STIM1 at the inclusion membrane when CRAC channel formation is induced and likely limits the amount of available STIM1 for formation of these channels. Some caution must be exercised in interpretation of these results since overexpression of STIM1 or Orai1 can dramatically increase the number of CRAC channels^40^. The stoichiometry of STIM1 to the *C. trachomatis* sequestering agent will thus be important when assessing the influence of *C. trachomatis* on SOCE since STIM1 binding to the inclusion membrane presumably lowers the available STIM1 for binding Orai1 and could lower the endogenous STIM1:Orai1 molecular ratio. Re-analysis of the data from Dzakah^41^ revealed that none of the isoforms of STIM1 or Orai1 were significantly up- or down-regulated at 20 h post chlamydia infection. As to whether or not STIM1 expressed at endogenous levels are able to form puncta in infected cells remains unclear.

The primary sites of *C. trachomatis* infection for genital infections in women and ocular infections are the endocervical epithelium and conjunctival epithelium, respectively^4^. As the first targets of infection, mucosal epithelial cells act as first responders to pathogen challenge by the secretion of cytokines and chemokines, and thus play a key role in the innate immune response^42^. In an immortalized primary human endocervix-derived epithelial cell line, a productive *C. trachomatis* infection mitigated a pro-inflammatory cytokine and chemokine response. Although the mechanism was not defined, it was proposed that the circumvention of a robust cytokine and chemokine response represented a potential evasion strategy promoting the establishment of a favorable intracellular niche within the endocervix epithelium^43^. SOCE impacts cytokine and chemokine signaling events^31^. The influence of *C. trachomatis*-mediated suppression of SOCE warrants further investigation.

The impairment to calcineurin activation and NFAT1 nuclear translocation demonstrates a specific signaling pathway that is affected by chlamydial suppression of SOCE. Although the major functions of NFAT are often considered in innate immune cells such as lymphocytes, macrophages, dendritic cells, or neutrophils, NFAT plays multiple additional roles in development and cellular differentiation^19^. NFAT is also expressed in multiple cell types^19^, including epithelial cells^44–46^ and endothelial cells^47^, however, direct information on how disruption of NFAT signaling might impact pathogen interaction with epithelial cells is limited. African swine fever virus inhibits NFAT-regulated transcription of immunomodulatory proteins by synthesis of a protein, A238L, that directly binds to and inhibits the calcineurin phosphatase activity required for NFAT activation^48^. Reactivation of latent Epstein-Barr virus to a lytic state via a Ca^2+^/calcineurin dependent activation of NFAT by a complex mechanism has been proposed to represent a negative feedback loop involving a viral protein, Zta, that directly binds to and attenuates NFAT^49^. *H. pylori* has the capacity to influence NFAT activity either positively or negatively by the bacterial proteins CagA or VacA, respectively, in gastric epithelial cells^46^. VacA forms an anion selective channel in the plasma membrane^50^, which is believed to deregulate membrane depolarization and inhibit SOCE. Although the mechanism of SOCE inhibition differs, *C. trachomatis* also inhibits SOCE and subsequent NFAT nuclear translocation. NFAT is a transcriptional regulator that, upon translocation to the nucleus, induces expression of several genes important in innate immune responses^51^. Presumably, inhibition of SOCE by chlamydiae and the resultant inhibition of NFAT translocation blocks transcription of NFAT regulated genes. We hypothesize that this inhibition of SOCE and NFAT signaling promotes chlamydial survival, possibly by downregulation of chemokine or cytokine production. Further studies in human primary cervical epithelial cells or animal model systems will undoubtedly be necessary to fully elucidate the benefits to chlamydial and host survival.

## Methods

### Bacterial and mammalian cell culture

HeLa 229 human cervical epithelial-like cells (American Type Culture Collection) were cultivated in RPMI-1640 (Gibco) supplemented with 5% fetal bovine serum (HyClone) at 37 °C and 5% CO_2_ in a humidified incubator. The *C. trachomatis* D (UW-3-Cx) and L2 (LGV 434) serovars were cultured in HeLa cells and EBs purified by density gradient centrifugation as previously described^52^. Infectious titers were determined by Inclusion Forming Unit (IFU) assays^53^ with inclusions detected by indirect immunofluorescence. Viability of infected and uninfected cells was determined by Trypan Blue exclusion.

### Cell transfection

For siRNA transfections, HeLa cells were seeded at 1 × 10^4^ cells/well in 96-well, black-wall, clear-bottom plates (Costar). Transfection complexes, either ON-TARGETplus Non-Targeting Control Pool siRNA (Dharmacon) or ON-TARGETplus SMARTpool STIM1 siRNA (Dharmacon) were diluted to a 1 µM working concentration in Opti-MEM. An equivalent volume of the diluted DharmaFECT 1 reagent and diluted siRNA were combined and incubated at room temperature for 30 min to form siRNA/DharmaFECT complexes. For the mock transfection, DharmaFECT 1 reagent in Opti-MEM was used. HeLa growth medium was exchanged with Opti-MEM while siRNA transfection complexes were generated. Following incubation, 0.1 mL of the diluted transfection complex was added per well. Cells were incubated for 48 h prior to cell infection.

For mammalian expression vector transfections, HeLa cells that were either infected with *C. trachomatis*, L2 for the stated times or uninfected were transfected with the desired mammalian expression vector using X-tremeGENE HP DNA transfection reagent (Sigma) according to the manufacturer’s instructions.

### Fura-2, AM Ca^2+^ measurement

HeLa cells were seeded in 96-well, black-walled plates at a density of 2 × 10^4^ cells/well. Cells were infected the following day with *C. trachomatis* serovar L2 at an MOI of 1. At the desired time post infection, medium was removed and cells were washed with PBS. Cells were loaded with 5 µM Fura-2, AM in Ca^2+^-containing Ringer’s Solution (140 mM NaCl, 5 mM KCl, 1 mM MgCl_2_, 10 mM HEPES, 2 mM CaCl_2_, 10 mM glucose, pH 7.4) for 1 h at room temperature. Cells were washed twice with PBS and incubated in Ca^2+^-free Ringer’s Solution (140 mM NaCl, 5 mM KCl, 1 mM MgCl_2_, 10 mM HEPES, 3 mM EGTA, 10 mM glucose, pH 7.4) for 30 min at room temperature. Following incubation, Fura-2, AM readings were performed by measuring fluorescence at 340/11 nm and 380/20 nm excitation and 508/20 emission every 20 s for 5 min using a Cytation 5 (BioTek). The solution was replaced with Ca^2+^-free Ringer’s solution containing either 2 µM thapsigargin (TG) or an equivalent volume of DMSO (vehicle control). Fluorescence readings were repeated every 20 s for 5 min. Each solution was then exchanged with Ca^2+^-containing Ringer’s solution, and fluorescence readings were taken every 20 s for 5 min. Each condition was performed in triplicate.

### Fluo-4, AM Ca^2+^ measurement

HeLa cells were seeded at 2 × 10^4^ cell/well in a 24-well, glass-bottom Cellvis imaging plate. The following day, HeLa cells were infected with *C. trachomatis* serovar L2 at an MOI of 1. At the desired time post-infection, cultures were washed with PBS and loaded with 2.5 µM Fluo-4, AM in Ca^2+^-containing Ringer’s Solution for 1 h at room temperature. Cells were washed twice with PBS and incubated in Ca^2+^-free Ringer’s solution (140 mM NaCl, 5 mM KCl, 3 mM MgCl_2_, 10 mM HEPES, 10 mM glucose, pH 7.4) for 30 min at room temperature. Following incubation, cells were imaged using the Nikon Ti2e with a CFI60 Super Plan Fluor Phase Contrast ADM ELWD 40 × Objective Lens (N.A. 0.6,; Nikon). ND acquisition (NIS-Elements 64-bit version 5.11.02 software (Nikon) was set to acquire images from 4 locations per well for infected and uninfected condition. ND acquisition was programmed to image every 30 s for 5 min. At the end of the Ca^2+^-free buffer imaging, the Ca^2+^-free buffer was exchanged with Ca^2+^-free buffer containing either 2 µM thapsigargin (TG) or DMSO carrier. Cells were imaged for another 5 min imaging interval. At the end of that interval, buffers were exchanged with a Ca^2+^-containing Ringer’s solution (140 mM NaCl, 5 mM KCl, 1 mM MgCl_2_, 10 mM HEPES, 3 mM EGTA, 10 mM glucose, pH 7.4), and images aquired for another 5 min interval. Huygens Essential software version 20.04 (Scientific Volume Imaging) was used to stabilize frames between time points and Imaris × 64 software version 9.6.0 (Oxford Instruments) was used to measure the mean fluorescence intensity of Fluo-4 per cell at each time point.

### GCaMP6m Ca^2+^ measurement

HeLa cells were seeded at 1 × 10^4^ cell/well in a 24-well, glass-bottom Ibidi imaging plate. The following day, HeLa cells were infected with *C. trachomatis* serovar L2 expressing mScarlet at an MOI of 1 and were transfected with pGP-CMV-GCaMP6m 4 h post-infection. The pGP-CMV-GCaMP6m plasmid was a gift from Douglas Kim and the GENIE project (Addgene plasmid # 40,754; RRID:Addgene_40754)^54^. At the desired time post infection, medium was removed from cells, and cells were washed with PBS. Cells were incubated in Ca^2+^-free Ringer’s solution for 15 min. Imaging, solution exchanges, image processing, and fluorescence measurements were performed as described in the Fluo-4, AM Ca^2+^ measurements section. Imaging was performed at 37 °C, 5% CO_2_, 92% relative humidity using a stage-top incubation chamber (Okolab).

### NFAT1-GFP nuclear translocation assay

Infections and transfections were performed as stated in the GCaMP6m Ca^2+^ measurement section, except that cells that were transfected with the HA-NFAT1(4-460)-GFP plasmid. The HA-NFAT1(4-460)-GFP plasmid was a gift from Anjana Rao (Addgene plasmid # 11,107 ;; RRID:Addgene_11107)^55^. At the desired time post infection, cells were washed twice with PBS, and then incubated in Ca^2+^ -free Ringer’s solution containing 1 ug/mL Hoechst stain for 30 min. Buffer was then exchanged with either DMSO or 2 µM TG Ca^2+^-free Ringer’s solution. Immediately following treatment, an image was acquired using the Nikon Ti2e with a CFI60 Super Plan Fluor Phase Contrast ADM ELWD 40 × Objective Lens. ND acquisition was programmed to acquire images from 4 locations per well for each condition. After incubating cells with either DMSO or TG for 5 min, the solution was exchanged with the Ca^2+^-containing Ringer’s solution, and then imaged at 18 min post Ca^2+^ addition. Imaging was performed at 37 °C, 5% CO_2_, 92% relative humidity using a stage-top incubation chamber (Okolab). Following imaging, Huygens Essential software version 20.04 (Scientific Volume Imaging) was used to stabilize frames between time points and Imaris × 64 software version 9.6.0 (Oxford Instruments) was used to measure the mean fluorescence intensity of NFAT-GFP in the nucleus and the cytoplasm of individual cells before and after SOCE induction. The NFAT-GFP N/C ratio was calculated as the mean nuclear NFAT-GFP fluorescence intensity ∕ the mean cytoplasmic NFAT-GFP fluorescence intensity.

### Transcriptional analysis of ORAI1 and STIM1

The RNAseq dataset from Dzakah et al. (PMID 33,397,284) was analyzed. The runs for uninfected or 20 hpi *C. trachomatis* serovar E infected samples were accessed through the Sequence Read Archive (BioProject PRJNA666640). Paired reads were mapped to STIM1 (Gene ID 6786), ORAI1 (Gene ID 84,876), and nine housekeeping genes^56^ using the Geneious read mapper (Geneious Prime version 2022.1.1). Expression levels were calculated for each gene simultaneously with ambiguously mapped reads counting as partial matches and transcripts were compared across all samples normalizing to the median gene expression ratios using the DESeq2 method. Significant differences in expression were determined as greater than 1 or less than − 1 log2 fold change with a *p*-value of < 0.05.

## Statistics

Statistical analysis was conducted using Prism version 9.1.1 software for Windows (GraphPad). An unpaired Student’s T test was performed for Fura-2, AM analysis, and Kruskal–Wallis test with Dunn’s posttest was performed for Fluo-4, AM relative change, GCaMP6m relative change, and NFAT-GFP N/C ratios. The datasets used and/or analyzed during the current study available from the corresponding author on reasonable request.

## Supplementary Information

Supplementary Information 1.

Supplementary Video 1.

Supplementary Video 2.

## Data Availability

The datasets used and/or analyzed during the current study available from the corresponding author on reasonable request.
