# Supplementary material for: Chlamydia trachomatis suppresses host cell store-operated Ca2+ entry and inhibits NFAT/calcineurin signaling
Source: Sci Rep. 2022 Dec 10;12:21406. doi: 10.1038/s41598-022-25786-y (PMC9741641; doi:10.1038/s41598-022-25786-y)

Extended Data

Extended Data Fig 1. Verification that STIM1 knockdown abrogates SOCE.

a A Fura-2, AM Ca^2+^ re-addition assay was performed using cells either transfected with non-targeting (N-T) siRNA or STIM1 siRNA. HeLa cells were either infected with *C. trachomatis* L2 for 24 hr or uninfected. b The relative change in [Ca^2+^]_i_ was calculated at the peak ER Ca^2+^ efflux induced by TG. Student’s T-test was used to compare the DMSO to the TG treatment, n=3. c The SOCE peak relative change in [Ca^2+^]_i_ was calculated. Student’s T-test was used to compare the uninfected TG treated condition to the 24 hpi TG treated condition, n=3. Data (a-c) are presented as mean ± SEM.

Extended Data **Fig 2. Fluo-4, AM analysis of *C. trachomatis* impairment of SOCE during late cycle development.**

**a** Single-cell analysis was performed with Fluo-4, AM-loaded HeLa cells either uninfected or infected with *C. trachomatis* L2 at 44 hpi and induced to undergo SOCE with TG. For each condition, ≥ 39 cells were analyzed. **b** Mean relative change in Fluo-4 fluorescence for uninfected and infected cells was calculated throughout the Ca^2+^ re-addition assay. **c** Single-cell analysis of the relative change in Fluo-4 fluorescence was calculated at the TG-induced peak. **d** Single-cell analysis of the relative change in Fluo-4 fluorescence was performed at the SOCE peak. A Student’s T-test was used for comparisons (**c,d**). Comparisons denoted with **** have a p value <0.0001 and ns represents no significant difference. Data (**b-d**) are presented as mean ± SEM.

**Extended Data Fig 3. GCaMP6m analysis of *C. trachomatis* impairment of SOCE at mid-late development.**

**a** Single-cell analysis of GCaMP6m-transfected HeLa cells was conducted for either uninfected or infected with *C. trachomatis* L2 at 36 hpi. For each condition, ≥ 24 cells were analyzed. **b** The mean relative change in GCaMP6m fluorescence was measured during Ca^2+^ re-addition assay. **c** Single-cell analysis of the relative change in GCaMP6m fluorescence was measured at the SOCE peak. A Kruskal-Wallis test was performed with Dunn’s post-hoc multiple comparisons test to compare conditions. Comparison denoted with **** has a p value <0.0001. Data (**b,c**) are presented as mean ± SEM.

**Extended Data Fig 4. *C. trachomatis* serovar D impairs SOCE.**

Live-cell microscopy of GCaMP6m was used to assess [Ca^2+^]_i_ changes within HeLa cells either uninfected or infected with *C. trachomatis* serovar D. For each condition, ≥ 69 cells were analyzed. **a** Single-cell analysis of relative change in GCaMP6m fluorescence was conducted throughout the Ca^2+^ re-addition assay. **b** The mean relative change in GCaMP6m fluorescence was measured from single cell analysis (**a**). **c** The relative change in GCaMP6m fluorescence at the TG peak was calculated for each condition. **d** The relative change in GCaMP6m fluorescence was assessed for the SOCE peak. Analyzes were performed using Kruskal-Wallis test with Dunn’s post-hoc multiple comparisons test (**c,d**). Comparisons denoted with **** have a p value <0.0001 and ns represents no significant difference. Data (**b-d**) are presented as mean ± SEM.

**Supplementary Information**

**Supplementary Video 1. Time series of NFAT-GFP nuclear localization in uninfected cells**

HeLa cells transfected with NFAT-GFP were treated with 2 µM TG in Ca^2+^-free Ringer’s solution. Following treatment, solution was exchanged with Ca^2+^-containing Ringer’s solution to induce SOCE. Time series was acquired by imaging cells every minute for 5 minutes in the Ca^2+^-free Ringer’s solution, and then every minute for 20 minutes in Ca^2+^-containing Ringer’s solution.

**Supplementary Video 2. Time series of NFAT-GFP nuclear localization in *C. trachomatis* infected cells**

HeLa cells were transfected with NFAT-GFP and infected with mScarlet-expressing *C. trachomatis* L2 for 24 hours. Cell treatments and imaging was performed as described in Supplementary Video 1. Asterisks indicate the nuclei of uninfected cells.

Extended Data Fig. 1


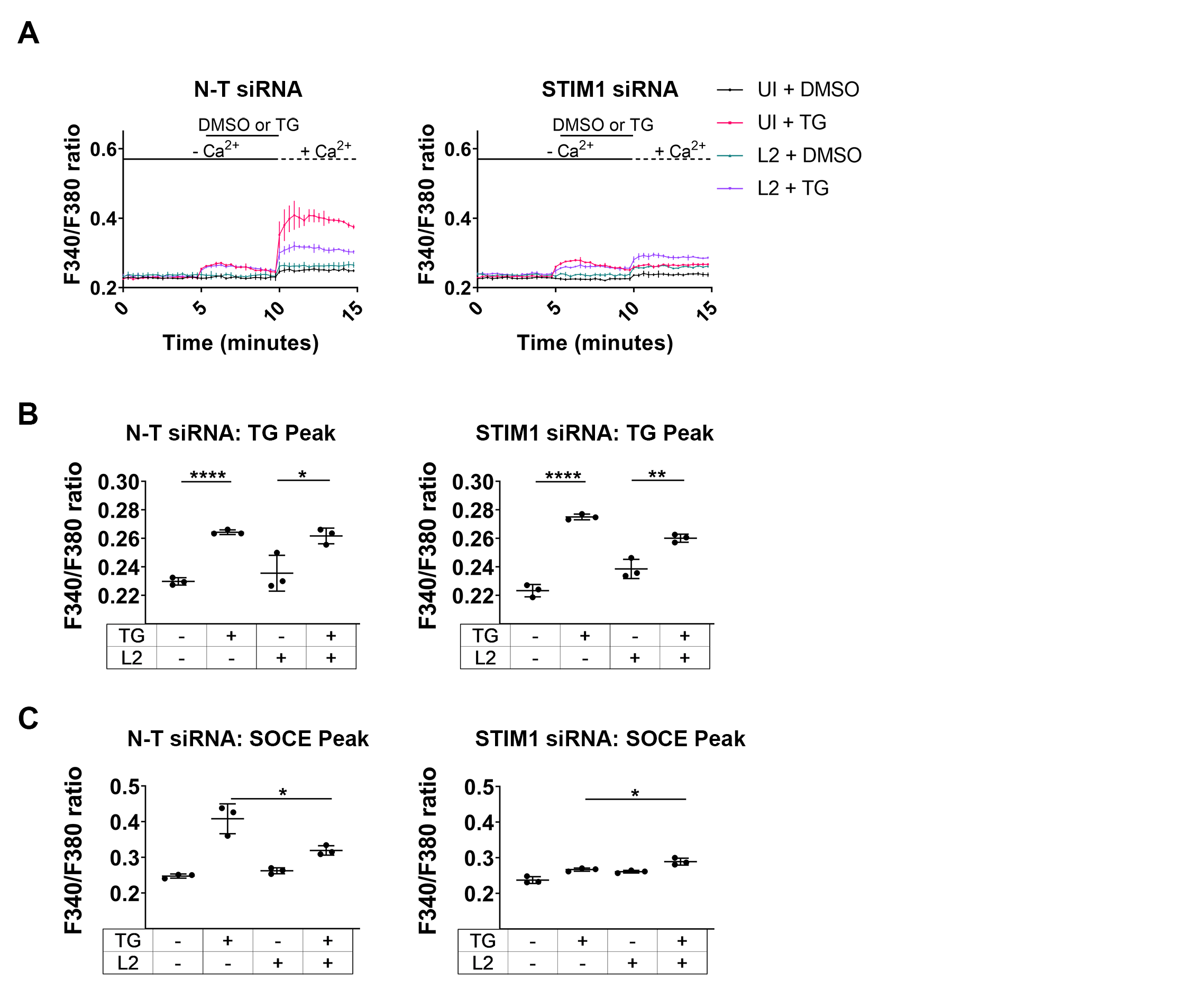


Extended Data Fig. 2


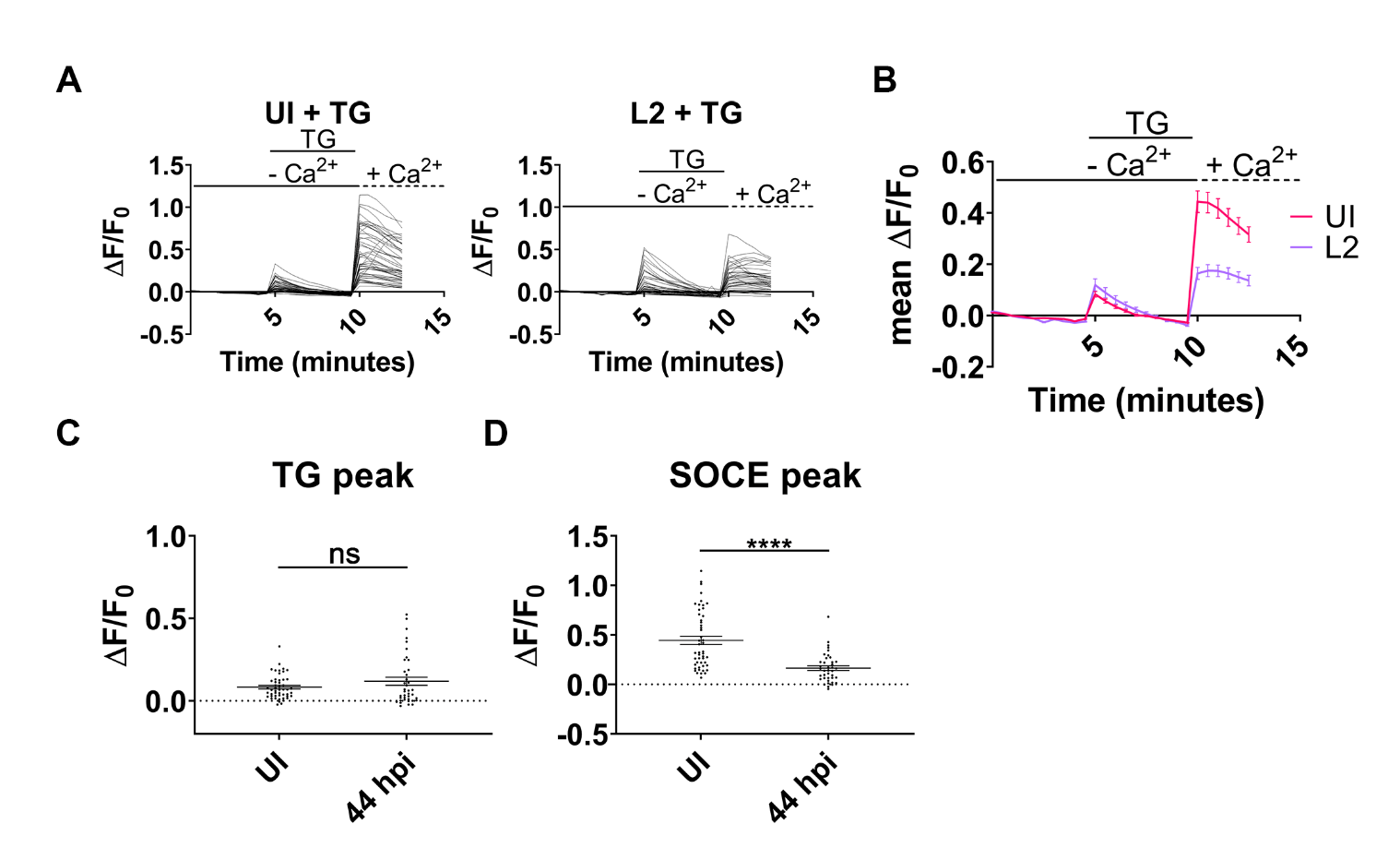


Extended Data Fig. 3


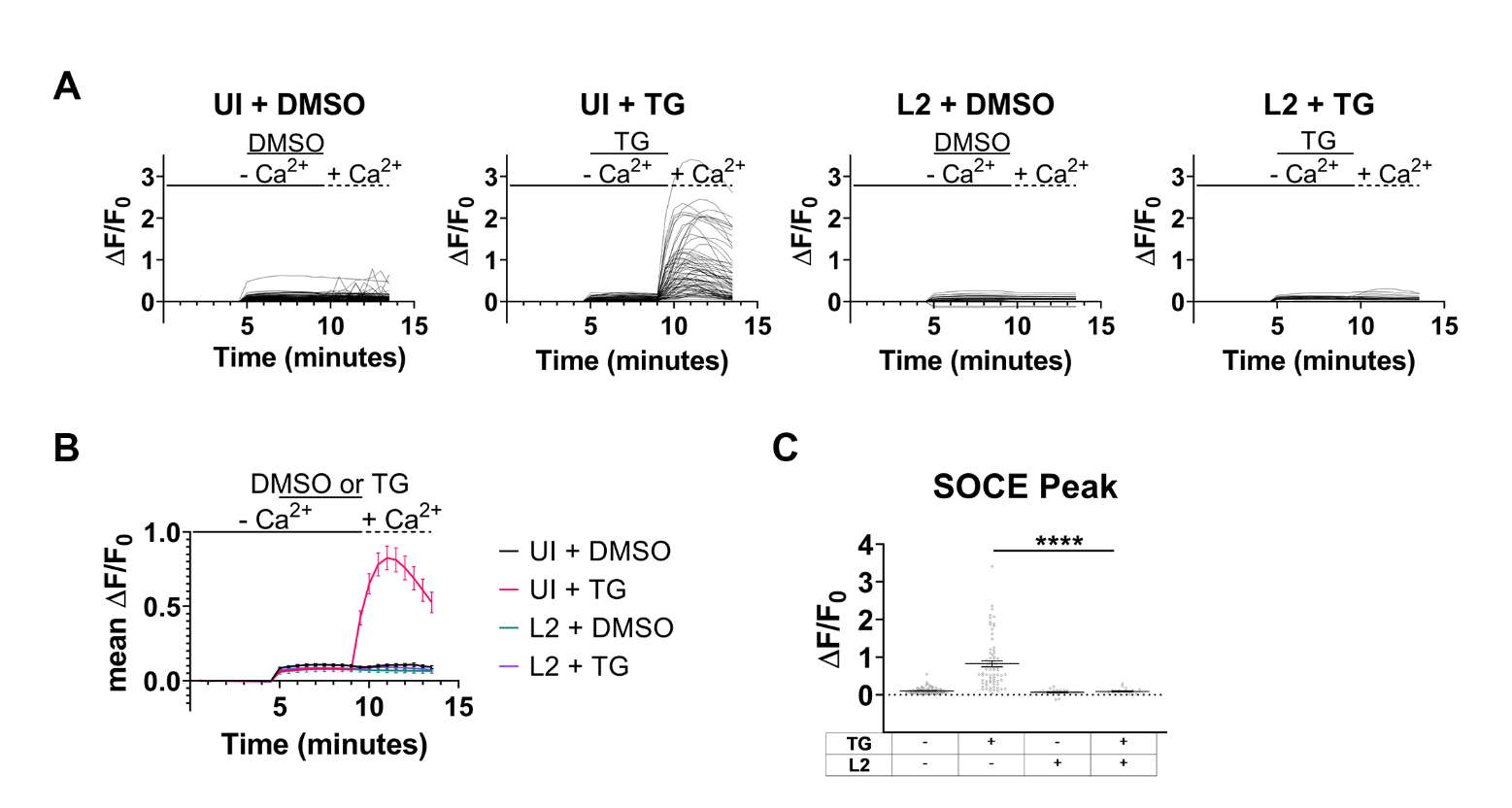


Extended Data Fig. 4


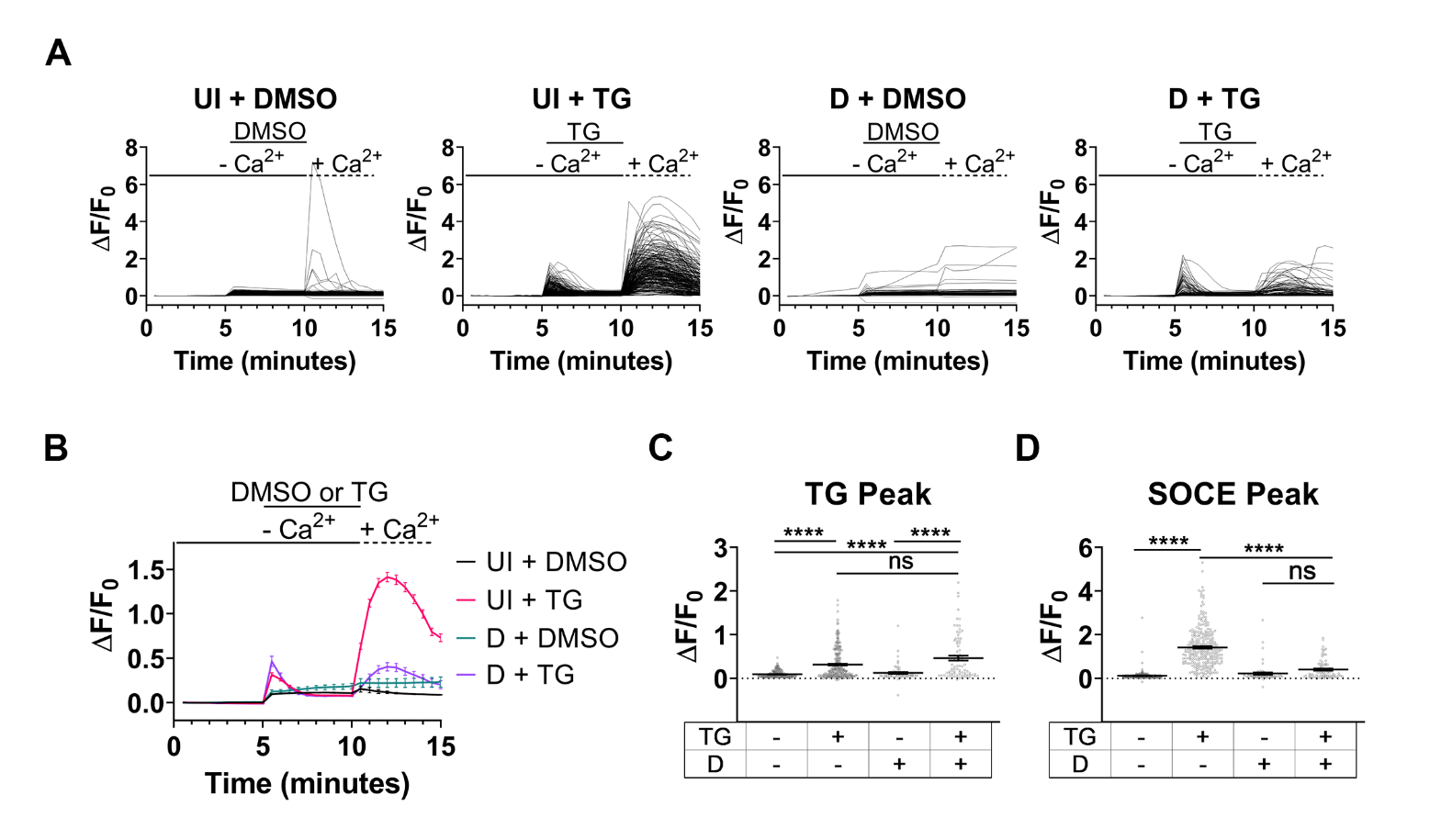

Supplement: Supplementary file 1 — Supplementary Information 1. [file 41598_2022_25786_MOESM1_ESM.docx]
